# The Association Between Lactational Infective Mastitis and the Microbiome: Development, Onset, and Treatments

**DOI:** 10.7759/cureus.62717

**Published:** 2024-06-19

**Authors:** Farishta Saifi, Benscar Jeoboam, Michelle Demory Beckler, Joshua M Costin

**Affiliations:** 1 Biomedical Sciences, Nova Southeastern University Dr. Kiran C. Patel College of Allopathic Medicine, Fort Lauderdale, USA; 2 Microbiology and Immunology, Nova Southeastern University Dr. Kiran C. Patel College of Allopathic Medicine, Fort Lauderdale, USA; 3 Medical Education, Nova Southeastern University Dr. Kiran C. Patel College of Allopathic Medicine, Fort Lauderdale, USA

**Keywords:** microbiome, entero-mammary pathway, prebiotics, probiotics, lactating, pregnancy, lactational infective mastitis

## Abstract

Lactational infective mastitis (LIM) was previously thought to occur due to trapped milk causing inadequate milk drainage and consequent infection. However, advances in genome sequencing techniques have shown that the abundance of *Staphylococcus aureus*, *Staphylococcus epidermidis*, *Lactobacilli* species, and *Bifidobacterium* species in the breast milk of lactating women play a key role in the development of LIM. Recent discoveries have revealed that the breast milk microbiome is composed of bacteria and other microorganisms, which are seeded through multiple pathways and are influenced by maternal factors. An imbalance in the microbial abundance in breast milk can lead to LIM. Given that this infection can cause early termination of breastfeeding, it is imperative to discuss prevention and treatment options. The objective of this review is to highlight the pathogens involved in LIM affecting human mothers, routes of bacterial transfer, and contributing factors that may influence changes in the composition of the milk microbiota, as well as propose preventative and curative treatment options.

## Introduction and background

Human breast milk was once widely considered sterile. However, in the past decade, certain types of bacteria have been found in the fresh milk of healthy, lactating women, suggesting that breast milk does indeed contain a commensal microbiota [1]. It is now accepted that healthy breast milk contains several protective nutrients, which serve as prebiotics, and microorganisms, which serve as probiotics and help create an environment for commensal gastrointestinal microbes in the infant’s gut. Globally, it is recommended that an infant should be exclusively breastfed during the first six months after birth [2]. Indeed, studies have indicated a link between a lack of infant gut microbiota diversity and metabolic disorders later in life, including obesity, diabetes, and cardiometabolic disorders [3]. Given that the composition of an infant’s gut microbiota can influence adult health, the diversity provided by breastfeeding is extremely important, and diseases that lead to early cessation of breastfeeding, such as mastitis, should be thoroughly examined.

Mastitis is defined as the inflammation of breast tissue and can be classified into two types: lactational mastitis and non-lactational mastitis. Lactational mastitis most commonly affects women during the first 6-12 weeks of breastfeeding and occurs in 7-10% of breastfeeding women in the United States [4]. The signs and symptoms of mastitis may vary depending on the cause, and diagnosis is generally made clinically, with patients typically manifesting localized, unilateral breast tenderness with redness of the skin, sore or cracked nipples, decreased output of breast milk, a fever of 101 °F, fatigue, body aches, and headache [5]. In some cases, a culture is collected to aid antibiotic therapy.

Lactational mastitis is commonly caused by trauma, genetic factors, immune factors, infant-feeding issues, and nutritional factors, as well as infection. When lactational mastitis is caused by infection, it is known as lactational infective mastitis (LIM). New evidence has emerged suggesting LIM can be caused by mammary dysbiosis [6]. Mammary dysbiosis is defined as a loss in the diversity of commensals due to an increased abundance of microbes with potential pathogenicity [7]. The presence of bacteria in milk seems to result from the mammary gland's colonization by multiple potential mechanisms, including translocation of maternal gut bacteria and retrograde milk flow [8-12]. Several maternal factors have been noted to influence the microbial composition of breast milk, such as weight during pregnancy, maternal diet, mode of delivery, gestational age, and maternal health [13-20]. Although *Staphylococcus* species are among the predominant commensal bacteria in the breast milk of healthy women, their concentrations are notably higher in breast milk samples taken from mothers with LIM [21]. In this review, we aim to discuss the link between LIM and the microbiome while exploring methods of preventative and curative treatments.

## Review

Bacterial translocation to maternal breast

Dysbiosis of bacteria in the breast ducts, glands, and milk may be involved in initiating and advancing LIM. However, the exact mechanism that introduces these microbes into the breast and breast milk is unclear. Two pathways are thought to be involved in bacterial colonization of breast milk during late pregnancy and lactation: the retrograde transfer and entero-mammary pathways. An ultrasound imaging study found that breast milk backflows from the human neonate's oral cavity into the mammary duct during suckling [8], suggesting that a neonate's oral cavity and breast skin may be a source of breast milk microbes. Additionally, breast milk samples taken after breastfeeding contained more *Streptococcus* species commonly found in neonatal oral cavities than before breastfeeding [9]. These studies strongly indicate that bacteria and possibly other microorganisms from neonates' oral cavities and breast skin can be transferred into milk ducts via the retrograde transfer pathway.

Alternatively, human pre-colostrum samples obtained from healthy first-time pregnant women before infant delivery showed the presence of strictly anaerobic bacteria primarily found in the human gastrointestinal tract, such as strains of *Bifidobacterium*, suggesting that there may also be an endogenous mechanism of microbial transfer from within the mother's body to breast milk [10]. Evidence of this gut-breast axis, now referred to as the entero-mammary pathway, was revealed when researchers found that pregnant mice had significantly higher levels of bacterial species in their mesenteric lymph nodes during late pregnancy and early lactation compared to control virgin mice, and 80% of the postpartum mice had viable bacteria within their mammary tissue 24 hours after delivery [11]. Furthermore, another study found DNA from gut-associated, obligate anaerobic bacteria in the stool of human mothers, their breast milk, and their neonates' stool [12], adding to the evidence of the entero-mammary pathway. 

**Figure 1:**
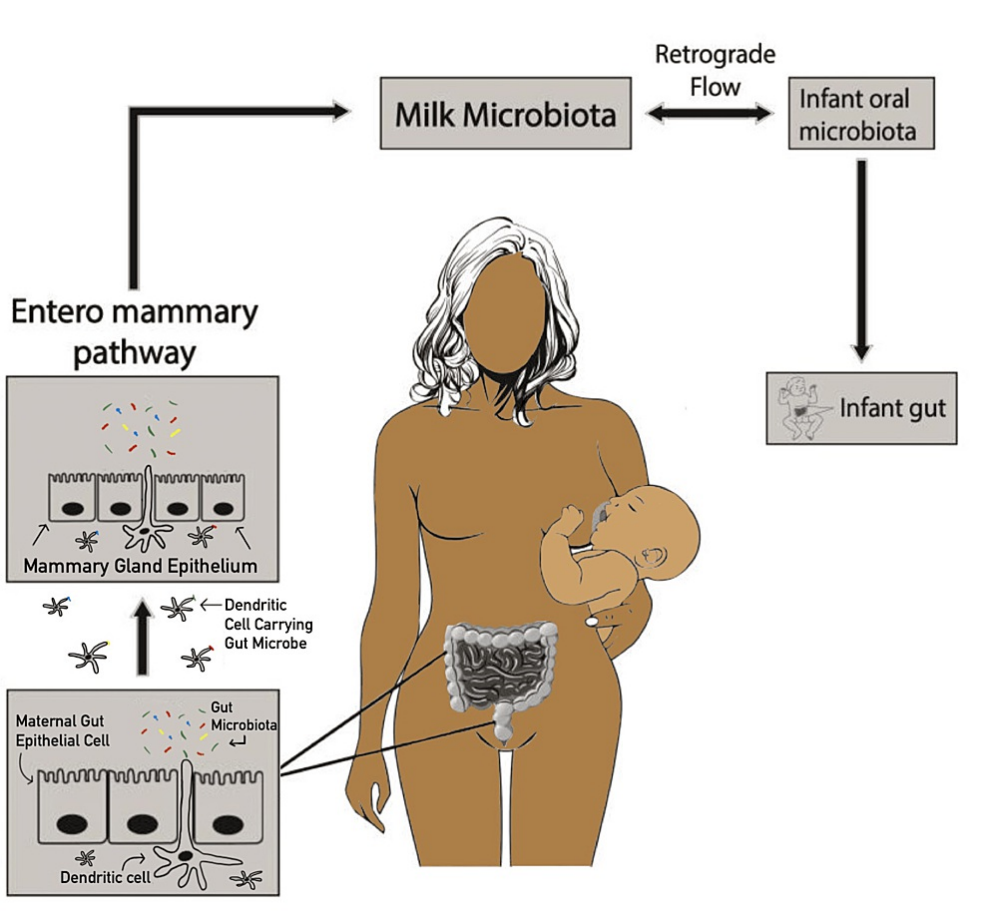
Maternal milk colonization pathways Image created by Benscar Jeoboam

The mechanism of the endogenous route that the bacteria travel to the breast milk is still unclear. However, studies indicate significant morphological and physiological changes during the last trimester of pregnancy and early lactation, which cause an increase in the outflow of leukocytes, such as dendritic cells, to migrate through tight junctions of intestinal epithelial cells and travel to mucosal sites [22]. Because dendritic cells can travel to different tissues and take up bacteria [23], it seems possible that there is movement of maternal gut bacteria through tight junctions of the intestinal mucosal layer to the mesenteric lymph nodes and then to the mammary glands, mediated by an elaborate interaction with gut immune cells, during late pregnancy and early lactation. Together, these data suggest but do not confirm a possible endogenous route of bacterial transfer from the gut to the breast. Future studies are needed to elucidate this possibility and the mechanism involved in bacterial transfer. Figure 1 illustrates the currently perceived process.

Breast milk microbes associated with LIM 

With both the retrograde transfer and entero-mammary pathway as possible mechanisms of microbial entry into mammary tissue, it seems plausible that these pathways serve as portals for numerous commensal bacteria, including those with pathogenic ability, to travel into mammary tissue and initiate dysbiosis, leading to LIM. Indeed, pregnant germ-free adult mice exhibited dysbiosis of intestinal microbiota followed by mastitis after fecal microbiota transplantation of bovine mastitic fecal samples [24], proving that circulating bacteria are involved in the development and onset of LIM. 

Three bacterial genera have been highly implicated in human LIM: *Staphylococcus, Lactobacillus*, and *Bifidobacterium*. Breast milk collected from acute LIM patients showed higher concentrations of *Staphylococcus aureus*, while subacute LIM patient samples had higher concentrations of *Staphylococcus epidermidis* than normal, healthy controls [25-27]. This data indicates that the opportunistic pathogens *S. aureus* and *S. epidermidis* significantly increase in the breast milk of LIM patients and can be referred to as LIM-causing agents. On the other hand, the number of *Lactobacilli *species and *Bifidobacterium* species appears to be decreased in the breast milk of LIM-suffering patients compared to breast milk from healthy controls [28,29]. Studies have shown that certain species of *Lactobacilli* and *Bifidobacterium* exhibit inhibitory activity against *S. aureus* and *S. epidermidis* in vitro [30-31], supporting the idea that it may be the lack of *Lactobacilli* and *Bifidobacterium* species which allows for the overgrowth of LIM-causing agents. By referencing this information, it appears that during LIM, the population of mammary opportunistic pathogens increases while commensal bacteria decreases, supporting the importance of microbiota dysbiosis in LIM. 

Several virulence factors, including toxin production and biofilm formation, are linked with antimicrobial resistance that may contribute to the onset of LIM by *S. aureus* and *S. epidermidis* [32,33]. In lactating women with mastitis, where *S. aureus* is the dominant bacteria, *S. aureus* proliferates in the mammary gland. It uses a collection of cell wall-anchored proteins to promote invasion and adhesion and avoid immunological defenses [34]. *S. aureus* also produces many toxins, including Staphylococcal enterotoxin C (SEC), which can exert pathogenic effects both as a superantigen and an antiangiogenic factor [35]. When SEC was injected into mouse mammary glands, mammary gland inflammation and severe infective mastitis resulted [36]. Therefore, it is likely that the ability of SEC to trigger inflammation, disrupt membrane permeability of mucosal cells, and activate T cells allows for an increase of bacterial translocation to the mammary gland, thus contributing to the onset of LIM. Future studies are needed to identify SEC-producing strains of *S. aureus* involved in human LIM and exactly how this toxin mediates the infective process. 

Biofilms have been recognized as an essential virulence factor amongst *Staphylococci* species in chronic mastitis infections because they are antimicrobial-resistant [37]. Within the narrow milk ducts, *S. aureus* and *S. epidermidis* can build biofilms and impair milk flow. This leads to increased macrophage activation, inflammation, and tissue damage, making the infection difficult to eradicate with antibiotic therapy alone. 

The role of alternative microorganisms in LIM

While most research has focused on the role of bacteria in LIM, it is possible that other microorganisms could facilitate the onset or progression of LIM by competing for the surrounding environment, host binding sites, and available nutrients [38]. A metagenomic study analyzing the microbiome of 20 human breast milk samples found differences in the presence of various microorganisms between healthy women and those with acute or subacute LIM [25]. *Malassezia-associated* DNA was found in all healthy breast milk samples but was absent in LIM samples. In addition, archaeal DNA was detected in healthy controls but lacking in all LIM samples. *Siphoviridae*, a known *Staphylococcus* phage that contributes to the pathogenicity of *S. aureus*, was detected in 7 of the 10 LIM samples but was utterly absent in healthy controls. Different parasitic organisms such as *Toxoplasma gondii *and* Giardia intestinalis* were detected in breast milk samples from healthy women and women suffering from mastitis without any clinical sign of infestation. Although the sample size was limited, this data suggests that the presence of certain fungi, archaea, viruses, and parasite species in breast milk may contribute to LIM. Past studies have shown that these microorganisms are essential to microbiome homeostasis [39-41]; however, future studies are needed to elucidate how the relative abundances of these microorganisms contribute to mammary microbiota dysbiosis and the pathogenesis of LIM. 

Maternal factors linked to LIM 

Several maternal factors have been identified as influencing the relative abundance of breast milk bacteria, particularly *S. aureus, S. epidermidis, Lactobacillus,* and *Bifidobacterium*, and may contribute to developing LIM. These factors include weight gain during pregnancy, diet, gestational age, mode of delivery, and health. 

Breast milk samples from mothers who experienced excessive weight gain during their pregnancy had higher counts of *Staphylococci* and *Lactobacillus* and lower counts of *Bifidobacterium* than from mothers who experienced normal weight gain [13,14]. Moreover, research suggests that maternal diet may play a role in developing mammary-related diseases [15], including LIM. Women with a diet consisting of high plant protein, fiber, and carbohydrates had higher breast milk microbial diversity, with significantly higher counts of *Staphylococcus, Bifidobacterium*, and *Lactobacillus* species as compared to breast milk from women with a diet consisting of high animal protein and lipids [16]. While this study did not account for food proportion sizes and daily habits such as exercise, stress handling, and home environment, it does appear that nutritional components can alter the mammary microbiome. 

Gestational age and mode of delivery can also influence the milk microbiome. *Bifidobacterium* concentration was significantly higher in gestation-at-term lactation compared to preterm gestation lactation [17]. Furthermore, breast milk samples taken from women who had undergone a cesarean section were found to be positive for *Lactobacillus* and *Bifidobacterium* DNA less frequently than in women who delivered vaginally. Since antibiotics are often administered to reduce the risk of infection associated with cesarean section, the differences seen in the breast milk microbiota were presumably also influenced by anti-biotherapy [18]. 

Maternal health plays a crucial role in pregnancy and birth-related outcomes. Breast milk from lactating women with celiac disease had lower *Bifidobacterium* species levels than milk from healthy women [19]. This result is particularly interesting given the role of mucosal barrier function in the entero-mammary pathway of microbial transfer. A breakdown in barrier function, as seen in inflammatory intestinal diseases, may cause the alteration of the milk microbiome by allowing increased movement of maternal gut bacteria through the intestinal mucosal layer to the mammary glands. Future studies should examine the association between other acute and chronic intestinal diseases and LIM. In addition, maternal postnatal psychosocial distress may also cause a change in milk microbial diversity. In milk samples taken from healthy women three months after full-term pregnancies, milk from women with low psychosocial distress had an increase in microbial diversity, which consisted of a significant decrease in abundance of Staphylococcus species and an increase in Lactobacillus species, compared to milk samples from women with high psychosocial distress [20]. 

While it remains unclear which maternal factors truly predispose to the onset of LIM, clinical observations suggest that women with a history of mastitis face a higher risk compared to those without such a history [4]. The presumed factors influencing milk microbiota are presented in Table 1.

**Table 1:** Maternal factors associated with bacterial diversity in breast milk

Maternal factors	Effect on breast milk bacteria abundance
Excessive weight gain during pregnancy	↑ Staphylococci bacteria
	↑ Lactobacillus
	↓ Bifidobacterium
A diet consisting of high plant protein, fiber, and carbohydrates	↑ Staphylococci bacteria
	↑ Lactobacillus
	↑ Bifidobacterium
Gestation-at-term	↑ Bifidobacterium
Vaginal birth	↑ Lactobacillus
	↑ Bifidobacterium
Celiac disease	↓ Bifidobacterium
High psychosocial distress	↑ Staphylococci bacteria
	↓ Lactobacillus

Treatment options for LIM

Traditionally, antibiotics effective against *S. aureus* are the most common treatment for LIM. These include beta-lactamase-resistant penicillin, such as dicloxacillin, cloxacillin, and flucloxacillin [42]. However, since multidrug resistance and the formation of biofilms are common features of *Staphylococcus* species [43], chronic and recurrent LIM can often occur. Equally important are the severe side effects of antibiotic use, such as the imbalance it can cause in the maternal intestinal and vaginal microbiome [44,45]. Furthermore, antibiotics have been shown to decrease the DNA levels of *Lactobacillus* and *Bifidobacterium* in breast milk [18,46]. This decrease is considered concomitant to an increase in pathogenic *Staphylococcus* species. Given the possible unfavorable, systemic effects of antibiotics on a woman's microbiome and, consequently, their neonates, alternative treatments for LIM could be advantageous. 

The oral administration of probiotics to pregnant and lactating mothers effectively prevents and treats LIM. Specific species of *Lactobacillus* and *Bifidobacterium* have been of particular interest as central components in probiotic supplements for the prevention and treatment of LIM due to their stimulation of the immune system, role in the development of the gut barrier function, bacteriocin-antimicrobial activities, and direct inhibition of strain-specific* Staphylococcus* virulence factors [47]. Oral probiotic regimens containing *L. salivarius *CECT5713 and *L. gasseri *CECT5714 given to women with LIM led to a significantly lower abundance of *S. aureus* and *S. epidermidis* in their breast milk and absence of clinical signs of mastitis compared to control [48]. Another study comparing the effectiveness of oral administration of probiotics versus antibiotic therapy for treating LIM found that women supplemented with *L. fermentum* CECT5716 or *L. salivarius* CECT5713 had significantly lower counts of *S. aureus* and *S. epidermidis* in their breast milk and reported greater alleviation of mastitis symptoms compared to the antibiotic group [49]. These two studies' results suggest that certain Lactobacilli strains may be used as an alternative treatment for infectious mastitis.

A follow-up study examined the possibility of preventing LIM by oral administration of *L. salivarius* PS2 during late pregnancy [50]. All participants in the study had been diagnosed with infective mastitis in a previous pregnancy and were assumed to be at risk for reinfection. An experimental group was given *L. salivarius* PS2 from gestational week 30 until delivery, and they were longitudinally evaluated during the first three months postpartum. The breast milk from women in the probiotic-mastitis group showed a decrease in *Staphylococcal *counts compared to breast milk from women in the placebo-mastitis group, resulting in a lower incidence of LIM in the probiotic group compared to the placebo group. A limitation of this study is the possibility of antibodies and immunity due to previous infection. Nevertheless, the results suggest that ingestion of *L. salivarius* PS2 during late pregnancy by mothers who previously had infective mastitis may help prevent a recurrence. In addition, a recent study showed that intramammary infusion of* Bifidobacterium breve *in dairy cows with chronic subclinical mastitis decreased somatic cell counts and mastitis-causing pathogens, including *S. aureus* [51]. More studies are needed to determine if intramammary infusion of probiotics can be a safe and effective treatment option for human mastitis. 

Inulin is a prebiotic dietary fiber that has been found to selectively promote the proliferation of strain-specific *Lactobacillus* and *Bifidobacterium* in the gastrointestinal tract of humans [52]. A study has found evidence suggesting that inulin supplementation may help treat LIM. In a study involving subclinical mastitic cows, the cows who were given inulin supplements for eight weeks had lower *Staphylococcus* milk counts, higher *Bifidobacterium* and *Lactobacillus* counts, and reduced inflammatory symptoms compared to the control group [53]. Given this result, it seems plausible that human supplementation of inulin may help reduce the symptoms of human LIM. However, in future studies, cross-feeding networks will be an essential consideration as more problematic bacteria, such as Enterobacteriaceae, have also been shown to be enriched by prebiotics [54]. Therefore, prebiotics may be contraindicated for some mothers, yet the benefits of prebiotics on symptoms of mastitis may outweigh the disadvantages. 

We postulate that modifying the oral microbiota of infants born to mothers with LIM using prebiotics and probiotics may work as a treatment option. This approach relies on the retrograde transfer pathway to transfer beneficial bacteria from the infant's oral cavity to the mother's breast milk. Infant formulas commonly contain prebiotic oligosaccharides to mimic the gut microbiota of a breastfed infant [55-57]. At the same time, probiotic supplementation with *Bifidobacterium* or *Lactobacillus* strains has been shown to colonize in the infant's gut beyond the supplementation period [58,59], potentially leading to long-term health benefits. As prebiotics and probiotics are considered safe for infants and can alter their gut and oral microbiome [60], exploring their use as a preventative and curative treatment option for LIM via retrograde transfer is warranted.

## Conclusions

LIM affects a significant proportion of women in the United States. The disease is much more complex than originally thought, and further studies should be performed to examine the impact of the relative abundances of microorganisms in breast milk and their virulence factors on the onset and progression of LIM. Manipulating the human breast milk microbiome via the entero-mammary pathway using prebiotic and probiotic supplements may be useful in preventing and treating LIM, given that they are generally safe to consume for pregnant and breastfeeding women. Overall, LIM appears to be a multifactorial disease, and prevention and treatment options should consider several maternal factors affecting the relative abundances of breast milk bacteria, including weight gain during pregnancy, diet, gestational age, mode of delivery, and overall health. The literature on the pathogens associated with LIM in human mothers is in its infancy, and the field needs to continue its focus on prevention and cure to help alleviate the discomfort in mothers with LIM and increase dedicated newborn-feeding time.
